# Uncovering students’ misconceptions by assessment of their written questions

**DOI:** 10.1186/s12909-016-0739-5

**Published:** 2016-08-24

**Authors:** Marleen Olde Bekkink, A. R. T. Rogier Donders, Jan G. Kooloos, Rob M. W. de Waal, Dirk J. Ruiter

**Affiliations:** 1 Department of Anatomy, Radboud University Medical Center, P.O. Box 9101, 6500 HB Nijmegen, Netherlands; 2 Department of Internal Medicine, Radboud University Medical Center, Nijmegen, Netherlands; 3 Department for Health Evidence, Radboud University Medical Center, Nijmegen, Netherlands; 4 Department of Pathology, Radboud University Medical Center, Nijmegen, Netherlands

**Keywords:** Misconceptions, Written questions, Student performance, Undergraduate medical education, Small group work, Gender differences

## Abstract

**Background:**

Misconceptions are ideas that are inconsistent with current scientific views. They are difficult to detect and refractory to change. Misconceptions can negatively influence how new concepts in science are learned, but are rarely measured in biomedical courses. Early identification of misconceptions is of critical relevance for effective teaching, but presents a difficult task for teachers as they tend to either over- or underestimate students’ prior knowledge. A systematic appreciation of the existing misconceptions is desirable. This explorative study was performed to determine whether written questions generated by students can be used to uncover their misconceptions.

**Methods:**

During a small-group work (SGW) session on Tumour Pathology in a (bio)medical bachelor course on General Pathology, students were asked to write down a question about the topic. This concerned a deepening question on disease mechanisms and not mere factual knowledge. Three independent expert pathologists determined whether the content of the questions was compatible with a misconception. Consensus was reached in all cases. Study outcomes were to determine whether misconceptions can be identified in students’ written questions, and if so, to measure the frequency of misconceptions that can be encountered, and finally, to determine if the presence of such misconceptions is negatively associated with the students’ course formal examination score. A subgroup analysis was performed according to gender and discipline.

**Results:**

A total of 242 students participated in the SGW sessions, of whom 221 (91 %) formulated a question. Thirty-six questions did not meet the inclusion criteria. Of the 185 questions rated, 11 % (*n* = 20) was compatible with a misconception. Misconceptions were only found in medical students’ questions, not in biomedical science students’ questions. Formal examination score on Tumour Pathology was 5.0 (SD 2.0) in the group with misconceptions and 6.7 (SD 2.4) in the group without misconceptions (*p* = 0.003).

**Conclusions:**

This study demonstrates that misconceptions can be uncovered in students’ written questions. The occurrence of these misconceptions was negatively associated with the formal examination score. Identification of misconceptions creates an opportunity to repair them during the remaining course sessions, in advance of the formal examination.

## Background

Pre-existing knowledge can positively influence how new concepts in science are learned [1, 2]. However, if new concepts conflict with pre-existing ideas, students may distort or ignore new information. Several terms are used in the literature to describe incorrect pre-existing ideas, including alternative conceptions, alternative frameworks and naïve beliefs. We use the term misconceptions throughout this article to describe students’ ideas that (1) are inconsistent with current scientific views [3], and (2) result in a misunderstanding or misinterpretation of new information [4]. Recognition of misconceptions is a highly challenging and difficult task for teachers as they tend to either over- or underestimate students’ prior knowledge [5]. Misconceptions are resistant to change [6] and can negatively influence students’ learning performance, which stresses the importance of identifying student misconceptions in order to achieve effective learning and teaching.

Misconceptions cannot be repaired unless they are recognized. Current teaching methods are not always effective in targeting and remediating misconceptions. Several studies demonstrated misconceptions prevailing throughout courses [6–9]. Current methods to test conceptual understanding and uncover misconceptions include: multiple choice questions (MCQs) with or without written explanations [4, 6, 10–17]; MCQs including a confidence test [18]; open questions [19]; generating MCQ questions by the student [20]; drawing [21] or selecting drawings [22]; individual interviews [21, 23]; laboratory instructions with or without (verbal) predictions of the outcome of the experiment [24]; online self-directed E-learning modules [25]; or in-depth interviews with teachers to explore their perceptions of student’s misconceptions [3].

MCQs are an efficient way to test large cohorts. However, a multiple choice questionnaire carries the disadvantage that students do not phrase or verbalize the misconceptions themselves, and, unfortunately, MCQs can inadvertently introduce new misconceptions. This occurs when students believe an incorrect alternative is correct. It is called a negative testing effect, and is aggravated when more false statements are included in a test [26]. Drawings provide a rich source of information about student thinking [21], but not all topics are suited to be expressed in drawings. Interviews are very successful in identifying misconceptions [21], but require substantial training of the interviewer, and are less efficient in large cohorts.

Each year, a large cohort of medical science and biomedical students enters our curriculum. Therefore we intended to explore an approach that is more efficient than interviews, but avoiding the risk of a negative testing effect by students adopting false answers, such as a multiple-choice questionnaire.

In a previous study [27] we investigated whether asking students to formulate written questions during small-group work sessions could enhance study performance. During subsequent evaluation of the questions we were struck by illogical and/or unclear elements in the formulations that reminded us of a misconception. Therefore, we wondered whether student’s written questions could be used to uncover misconceptions.

Formulating questions could be educationally relevant for several reasons. Asking questions: (1) stimulates critical thinking [28]; (2) stimulates students to focus on the issues to be studied [29, 30]; (3) forces them to reflect on their learning [31]; (4) provides information on the progress of the learner [20]; and (5) enhances the dialogue among students [32]. Writing down questions forces students to focus and formulate in a clear and concise way. The current explorative follow-up study was conducted to explore the following approach: challenging students to formulate written open questions, which were subsequently evaluated by experienced tutors in order to uncover misconceptions. Based on our experiences in a previous study [27] the current study was designed in the context of a small-group work session, as this was considered a highly suitable environment to challenge individual students to formulate written questions because of the safe learning environment, and the small-scaled setting for dialogue. In this small-scaled setting, students are constantly testing their mental models through interactions with one another and with the tutor [33]. The students are actively engaged in the learning process, which enhances their conceptual understanding, based on the constructivist theory of learning [34, 35]. To the best of our knowledge, challenging students to formulate written questions during SGW has not yet been used to detect their misconceptions. Therefore, the aim of this study was i) to determine whether misconceptions can be uncovered in students’ written questions, and if so, ii) to measure the frequency of misconceptions that can be detected in this particular setting. In addition, iii) the difference in the number of misconceptions according to gender and discipline of the students was assessed. Finally, iv) it was determined if the presence of such misconceptions is negatively associated with the students’ course examination results.

## Methods

### Participants and setting

The study was conducted during a second-year bachelor course on General Pathology at the Radboud University Nijmegen Medical Centre, the Netherlands, taken by 397 students from the medical and biomedical science discipline. A learner outcome-oriented curriculum consisting of consecutive courses was provided in which each course lasted 4 weeks. The successive topics of the course on General Pathology were: (1) Principles of Diagnosis and Cellular Damage; (2) Inflammation and Repair; (3) Circulatory Disorders; and (4) Tumour Pathology (pathogenesis and progression). Each topic had a consistent sequence of educational activities: lecture (voluntary); task-driven self-study in preparation for the subsequent SGW; SGW (voluntary); practical course (obligatory); interactive lecture (voluntary); and non-directed self-study. The study was executed during the voluntary SGW session on the topic of Tumour Pathology (2 h) during the 4th week. These sessions involved groups of 12–15 students. On the final day of the course, students were subjected to a formal examination on all four topics.

### Procedure

At the start of the SGW on Tumour Pathology, the tutor invited the students to think about an extra question related to the topic. This aimed at a question on disease mechanisms (conceptual understanding) and not mere factual knowledge. Tutors used a guided instruction to invite the students (Appendix). Students were told that they were provided questions in their manual to guide the discussion, but that they were challenged to come up with one additional open question themselves to stimulate the discussion even further. They were told it could be a question that represented a difficult issue for the student, or an issue that they would like to discuss further, eg during the subsequent interactive lecture. Students did not have to provide answers. At the end of the SWG, students wrote their individual question about the topic.

Questions were assessed by two independent expert pathologists (DJR, RdW) who were blinded to the students’ gender and discipline. The operational definition used to recognize a misconception was: an illogical or unclear presupposition incongruent with the current state of scientific knowledge/ professional standard. Knowledge gaps were not classified as misconception, but were considered a result of insufficient preparation to the SGW session. If the expert pathologists did not agree initially on whether or not a question contained a misconception, a third expert pathologist (ES) discussed the question with the other two experts. Consensus was reached in all cases. Questions including grammatical errors making them impossible to interpret, and questions that were not original (e.g copied from the students’ course manual) were excluded. Questions derived from students who did not attend the formal examination were also excluded.

### Study outcomes

The primary study outcome was to determine whether misconceptions can be uncovered in students’ written questions. Subsequent outcome measures were: the percentage of questions containing a misconception; the observed agreement among independent raters; the difference in the number of misconceptions among male/female students and medical/ biomedical students; and the formal examination score on Tumour Pathology and the remaining topics of the course: Principles of Diagnosis and Cellular Damage; Inflammation and Repair; and Circulatory Disorders. The formal examination score of the studied topic Tumour Pathology was compared to the score of the other three topics. In this way it was explored if students holding misconceptions generally performed lower in all course examination topics, or whether there was a topic-specific underperformance.

### Statistical analysis

Linear mixed models with an SGW-group-dependent random intercept were used in order to account for the dependence caused by clustering of the students into SGW groups [36]. After the primary analysis, subgroup analyses were performed according to gender and discipline. Cohen’s kappa was used to determine inter-rater agreement.

## Results

### Participation

**Fig. 1:**
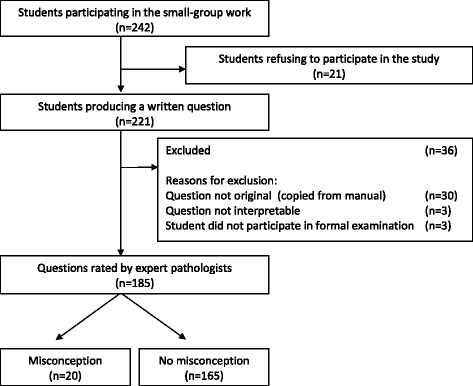
Flow chart assessment of questions

A total of 242 students attended the voluntary SGW sessions. In all, 221 students from the SGW group agreed to formulate a written question. Participation rate among the students in the SGW group sessions was 91 %. A total of 36 students were excluded because their questions were copied from the course manual (*n* = 30), not interpretable (*n* = 3), or because the student did not attend the formal examination (*n* = 3) (Fig. 1). A total of 185 students were included in the study: 132 female and 53 male students, 160 medical and 25 biomedical students.

### Misconceptions

Of the 185 questions rated, 11 % (*n* = 20) was classified as a misconception. The observed agreement among independent raters was 0.91 (95 % confidence interval [CI] 0.86–0.95), Cohen’s kappa: 0.51 (95 % CI 0.30–0.72). Inter-rater agreement was considered moderate. Examples of written questions containing a misconception are shown in Table 1. There was no difference in the prevalence of questions containing misconceptions among male and female students. All questions containing misconceptions were derived from medical students; questions written by biomedical science students did not reveal misconceptions.

**Table 1:** Examples of questions containing a misconception

Student’s question	Pathologist’s comment
Can cancer form leukaemia as a metastasis?	Leukaemia is a form of cancer that is not formed from a solid tumour. Since leukaemia is, in itself, already diffusely spread, the term ‘metastasis’ is inappropriate.
How does an HPV virus implement its RNA in the cell’s nucleus?	HPV is a DNA virus. Its DNA may be inserted in the cell’s DNA. With RNA as such, this is impossible.
How do benign lesions of the uterine cervix influence tumour suppressor genes?	In benign lesions, tumour suppressor genes function in the normal cell cycle by regulating cell growth and differentiation. Cells cannot influence these genes to become abnormal.

### Formal examination scores

Formal examination score on Tumour Pathology amounted to 5.0 (SD 2.0) in the group with misconceptions and 6.7 (SD 2.4) in the group without misconceptions (*p* = 0.003). The average formal examination score on the other topics of the course, including: (1) Principles of Diagnosis and Cellular Damage; (2) Inflammation and Repair; and (3) Circulatory Disorders, was not significantly different: 6.9 (SD 0.95) in the group with misconceptions versus 6.9 (SD 1.1) in the group without misconceptions (Table 2).

**Table 2:** Formal examination scores

	Formal examination score on Tumour Pathology and the remaining three topics of the course 1–10 scale (SD)		
Topic	Students with questions containing misconceptions	Students with questions without misconceptions	*P* value
Tumour Pathology	5.0 (2.0)	6.7 (2.4)	0.003
Other topics^a^	6.9 (0.95)	6.9 (1.1)	n.s.

^a^Other topics include (1) Principles of diagnosis and cellular damage; (2) Inflammation and repair and (3) Circulatory disorders

*n.s*. not significant

## Discussion

### Summary of the main findings

Students’ written questions can be used to uncover their misconceptions, ie in 11 % of the questions evaluated. The presence of such misconceptions was negatively associated with their course examination score. Students holding misconceptions in Tumour Pathology do not perform lower in the other course examination topics compared to students without misconceptions, but only in tumour pathology, which implies a topic specific underperformance. There was no association between the number of misconceptions and gender. Surprisingly, all misconceptions were identified in questions posed by students from the medical discipline; biomedical science students posed no misconceptions. The possible reason for this will be discussed later.

### Strengths of the present study

To the best of our knowledge this is the first prospective cohort study to assess students’ written open questions as an approach to identify misconceptions. The study was executed in a large cohort of students, which can be considered a strength, as it can be difficult to identify misconceptions among individual students in large cohorts [18]. Expert pathologists, all experienced tutors, independently executed a careful evaluation of open questions in order to uncover misconceptions.

Timely detection and correction of misconceptions is essential in learning environments based on the constructivist theory of learning in which students construct knowledge by appreciating new concepts in the context of their prior knowledge [37]. Construction and reconstruction of mental models is considered a central element of active student centered learning [38]. As Dennick stated, the constructivist theory implies that activation of prior knowledge may reveal incorrect conceptual understanding [37]. Challenging students during SGW to formulate a written question as demonstrated in this study seems a potential approach to expose students’ conceptual misunderstanding. In addition, writing questions forces students to focus on uncertainties and to formulate concisely. This may stimulate deep learning as students are applying their mental models using the new information that has recently been taught and discussed during the SGW.

### Limitations of the present study

An accurate interpretation of written questions is not an easy task, as reflected by the Cohen’s Kappa being moderate. Judgement could be enhanced by asking students to provide answers to their questions, which could give more information on student’s understanding. The current study primarily focused on identification of misconceptions as a first step of a series of activities to identify and remediate misconceptions. The most effective way of remediation followed by assessment of persisting misconceptions on the long term is to be investigated. The current outcome measures do not allow assessment of resistance of the misconceptions, as a specific follow-up survey was not part of the current study.

Selection bias may have occurred, as participation in the SGW session was not mandatory. This could possibly have resulted in selection of the more motivated students. High-achieving students with a higher degree of intrinsic motivation might pose fewer questions containing a misconception. The difference in misconceptions between medical and biomedical science students could reflect the extended background in science methodology of biomedical science students. During their training, more emphasis is given to scientific questioning, in comparison with medical training. However, the difference could also be explained by selection bias, which could be assessed by replication of the study during an obligatory SGW session.

### Comparison to the literature

There is an extensive body of research available on misconceptions, especially in the field of physiology. Sircar and Tandon conducted an observational study using written questions by students to induce in-depth learning and identify misconceptions [20]. In contrast to our study, Sircar and Tandon used MCQs instead of open questions, and provided a more competitive environment. They observed that posing questions led to lively discussions among students in tutorial classes, and that the written questions revealed misconceptions, although the prevalence was not reported.

Curtis et al. investigated misconceptions among dental students, and found the group of students with the lowest scores on the test to be similar to the group of students with the most misconceptions, although not completely identical [4]. Furthermore, this study was congruent with ours in the fact that there was no difference reported between male and female students with respect to the percentage of misconceptions. Badenhorst et al. conducted a qualitative study among teachers using in-depth interviews to explore their perceptions of student’s misconceptions [3]. Several misconceptions were reported, including those related to learning styles, as passive learners just absorb information without seeking for coherence. This stresses the importance of testing students’ conceptual understanding, because students seem to understand less than they appear to know [6, 10]. Students can give the right answers to MCQ tests based on correctly memorized facts without having developed a conceptual understanding of the disease mechanisms, making them unable to construct the right answer based on their mental model [39]. This poses a threat to meaningful learning, because the half-life of newly acquired knowledge is short if the students do not understand why their answers are correct.

### Implementation in practice: (1) misconceptions inventory

Evaluation of open questions by three expert pathologists is time consuming. Therefore, possible implementation in practice requires careful consideration in terms of the intended purpose. We see two different purposes for the approach demonstrated in this study. The first is to create an inventory of the existing misconceptions within the theme. A scrutinized assessment of the questions by expert pathologists is needed to serve this purpose. The list of misconceptions can be clustered in a ‘misconceptions inventory’. Such an inventory can be disseminated among tutors, so that they can challenge students to elaborate on these difficult topics to improve teaching and learning during subsequent courses. Especially less experienced tutors could benefit from using the misconceptions inventory that was created, based on other tutors’ experiences, to prepare their teaching activities.

### Implementation in practice: (2) using students’ written questions to feed the dialogue

The second purpose of our approach is to encourage dialogue among students. To serve this purpose, students’ written questions could be rotated among their peers in the small working group. Students could be asked to assess their peers’ written questions, search for misconceptions, and discuss these in small groups, in order to feed their dialogue and have students elaborate on their thinking. This approach is not time consuming for tutors and is suitable for application in large cohorts.

### Once misconceptions are uncovered: implications for future studies

It is obvious that identifying misconceptions alone is not enough to resolve them. Identification should be followed by remediation. Merely telling the student that their conceptual understanding is incorrect is unlikely to have effect. Students are to be challenged to test their mental models and experience that applying their incorrect beliefs results in incorrect answers. Reparation of misconceptions during an ongoing course could be executed during interactive sessions such as such as small group sessions and interactive lectures. During such an interactive session students can be engaged in a lively structured dialogue with their peers and with the tutor whereby their faulty mental models can be reconstructed [40]. The misconceptions can be used as input for the dialogue and evoke in-depth discussion among students. Future research could be directed to finding the most effective way to accomplish successful reparation. As misconceptions can be resistant to change [6] these follow up studies should preferably include repeated measurement of misconceptions on the long-term to assess the effectiveness of remediation.

## Conclusions

This study demonstrates that misconceptions can be uncovered by analyzing students’ written questions. The occurrence of these misconceptions is negatively associated with the formal examination score, which supports the idea that misconceptions interfere with effective student learning.

This approach can be useful in confronting students with their misconceptions and provides an opportunity to discuss and correct them during subsequent interactive sessions of the course in advance of the formal examination.

## Appendix

### Tutor instruction to be used during the small group session on tumour pathology

At the start of the small group session, please announce the following:

The goal of this small group session is to provide the opportunity to discuss the subject tumour pathology.

To guide this discussion a couple of questions are listed in your manual. To encourage the discussion, we would like to challenge everyone to formulate an additional question on the topic tumour pathology at the end of this small group session.

For this we would like to propose:

- Please, think about the question individually.
- Please, formulate a deepening question relating to disease mechanisms. Please avoid questions that are purely focused on factual knowledge.
- Ten minutes before the closure of the small group session you will be handed a form. Please write down your question and student number.
- Afterwards please discuss the questions plenary and pick the two most relevant questions. This can be determined based on consensus or voting. You can pick a question that is still unclear to you, or a question that has already been discussed. It should preferably be a question that is relevant to your fellow students (participating in the parallel small group sessions).
- Please hand in all the forms to the tutor.

## Abbreviations

SGW: Small group work
